# A framework for more equitable, diverse, and inclusive Patient and Public Involvement for palliative care research

**DOI:** 10.1186/s40900-023-00525-3

**Published:** 2024-02-08

**Authors:** Sarah Mitchell, Nicola Turner, Kate Fryer, Jude Beng, Margaret E. Ogden, Melanie Watson, Clare Gardiner, Joanne Bayly, Katherine E. Sleeman, Catherine J. Evans

**Affiliations:** 1 Division of Primary Care, Palliative Care and Public Health, Leeds Institute of Health Sciences, University of Leeds, Clarendon Road, Leeds, UK; 2 School of Health Sciences, Queen’s Medical Centre, University of Nottingham, Nottingham, UK; 3 Academic Unit of Primary Medical Care, University of Sheffield, Herries Road, Sheffield, UK; 4 Policy and Rehabilitation, Florence Nightingale Faculty of Nursing, Midwifery and Palliative Care, Kings College London, Cicely Saunders Institute of Palliative Care, London, UK; 5 Health Sciences School, University of Sheffield, 3a Clarkehouse Rd, Sheffield, UK; 6 St Barnabas Hospices, Worthing, UK

**Keywords:** Patient and Public Involvement, Patient engagement, Inequalities, Primary care, Palliative care, Equity, Equality, Diversity, Inclusion

## Abstract

**Background:**

There are marked inequalities in palliative care provision. Research is needed to understand how such inequalities can be addressed, so that everyone living with advanced illness can receive the care they need, when they need it. Research into inequalities in palliative care should be guided by Patient and Public Involvement (PPI) that includes people from diverse backgrounds, who are less likely to receive specialist services. Multi-disciplinary research partnerships, bringing together primary care (the main providers of palliative care to diverse communities) and specialist palliative care, have the potential to work together in new ways to do research to address inequalities and improve palliative care in practice. This report describes a research partnership between primary care and palliative care that aimed to: (1) create opportunities for more inclusive PPI in palliative care research, (2) co-design new resources to support more equitable, diverse and inclusive PPI for palliative care, (3) propose a new framework for inclusive PPI in palliative care research.

**Methods:**

PPI members were recruited via primary care and palliative care research networks from three diverse areas of the UK. A pragmatic, collaborative approach was taken to achieve the partnership aims. Online workshops were carried out to understand barriers to inclusive PPI in palliative care and to co-design resources. Evaluation included a “you said, we did” impact log and a short survey. The approach was informed by good practice principles from previous PPI, and existing theory relating to equity, equality, diversity, and inclusion.

**Results:**

In total, 16 PPI members were recruited. Most were White British (*n* = 10), other ethnicities were Asian (*n* = 4), Black African (*n* = 1) and British mixed race (*n* = 1). The research team co-ordinated communication and activities, leading to honest conversations about barriers to inclusive PPI. Resources were co-designed, including a role description for an Equity, Equality, Diversity and Inclusion Champion, a “jargon buster”, an animation and an online recipe book (http://www.re-equipp.co.uk/) to inform future PPI. Learning from the partnership has been collated into a new framework to inform more inclusive PPI for future palliative care research.

**Conclusion:**

Collaboration and reciprocal learning across a multi-disciplinary primary care and palliative care research partnership led to the development of new approaches and resources. Research team commitment, shared vision, adequate resource, careful planning, relationship building and evaluation should underpin approaches to increase equality, diversity and inclusivity in future PPI for palliative care research.

**Supplementary Information:**

The online version contains supplementary material available at 10.1186/s40900-023-00525-3.

## Plain Language Summary

Research is needed to understand how inequalities in palliative care can be addressed, so that everyone living with advanced illness can receive the care they need. Research into inequalities in palliative care should be guided by Patient and Public Involvement (PPI) that includes people from diverse backgrounds, who are less likely to receive specialist palliative care. Primary care services are grounded in the community they serve and can be the main providers of palliative care, but this is rarely the focus of research. Primary care and palliative care researchers can work together in new ways to do research to address inequalities and improve palliative care in practice. This paper describes the work of the RE-EQUIPP (REducing inEQUalities through Integration of Primary and Palliative Care) Care Partnership. The partnership involved researchers from primary care and palliative care working with people with lived experience of serious illness as patient or carer from three diverse areas of the United Kingdom: (1) London, (2) inner-city Sheffield and (3) Worthing in Sussex, a rural, coastal setting. The project provided opportunity to develop new ways of working and resources for more inclusive and equitable PPI for future palliative care research. Sixteen PPI members from diverse backgrounds and with a range of experience joined the partnership. Workshops were held to understand the barriers to inclusive PPI. New roles and resources were developed, including an Equity, Equality, Diversity and Inclusion Champion role, a “jargon buster”, an animation, and an online recipe book to inform future PPI. Learning from the partnership was used to develop a new framework, which is presented to inform inclusive PPI for palliative care research in the future. This outlines the need for research team commitment and shared vision, adequate resource, careful planning, relationship building and evaluation.

**Supplementary Information:**

The online version contains supplementary material available at 10.1186/s40900-023-00525-3.

## Background

Patient and Public Involvement (PPI) is defined as the active involvement of patients and members of the public in research prioritisation, design, and processes. It aims to ensure that research is relevant, contextual, inclusive and widely acceptable [1]. There are longstanding inequalities in palliative care. People with noncancer conditions, from areas of high socioeconomic economic deprivation, and from minority ethnic backgrounds are less likely to receive specialist palliative care services [2–5]. The delivery of palliative care to diverse communities by primary care is under-researched. Multi-disciplinary, primary and palliative care research partnerships have the potential to work together in new ways to improve inclusivity in PPI and conduct research to address inequalities.

As in research more widely, barriers to meaningful PPI in palliative care include mixed engagement amongst researchers, power dynamics in interactions and managing emotions, which can hamper efforts to effectively engage with, and learn from, people with lived experience from diverse groups [6, 7]. There is a risk of unconscious bias and tokenism [8]. A lack of opportunity for people from diverse and under-represented backgrounds to participate in PPI and research is reflective of the Inverse Care Law [9]. This proposes that populations most in need of good medical care are the least likely to receive it, because of challenges in availability and access to services [10].

This paper reports new approaches and resources to improve equity, diversity and inclusion in PPI developed during a research partnership project funded by the National Institute of Health Research (NIHR) for 12 months (2022), the RE-EQUIPP Care (REducing inEQUalities through Integration of Primary and Palliative Care) Partnership. The project was carried out following the COVID-19 pandemic, during a cost-of-living crisis in the UK, when health inequalities and population need for good palliative care were both rising [11, 12]. Throughout the report, Patient and Public Involvement (noun) is abbreviated to “PPI”. The term “PPI members” describes patient and public partners and was agreed to promote a sense of equal partnership and belonging. This paper reports patient and public involvement, not a research study.

## Methods

### Aims

The aims of the project were to:

1. Create opportunities for more inclusive PPI in palliative care research,
2. co-design new resources to support more equitable, diverse, and inclusive PPI for palliative care
3. propose a new framework for inclusive PPI in palliative care research.

### Setting

**Fig. 1:**
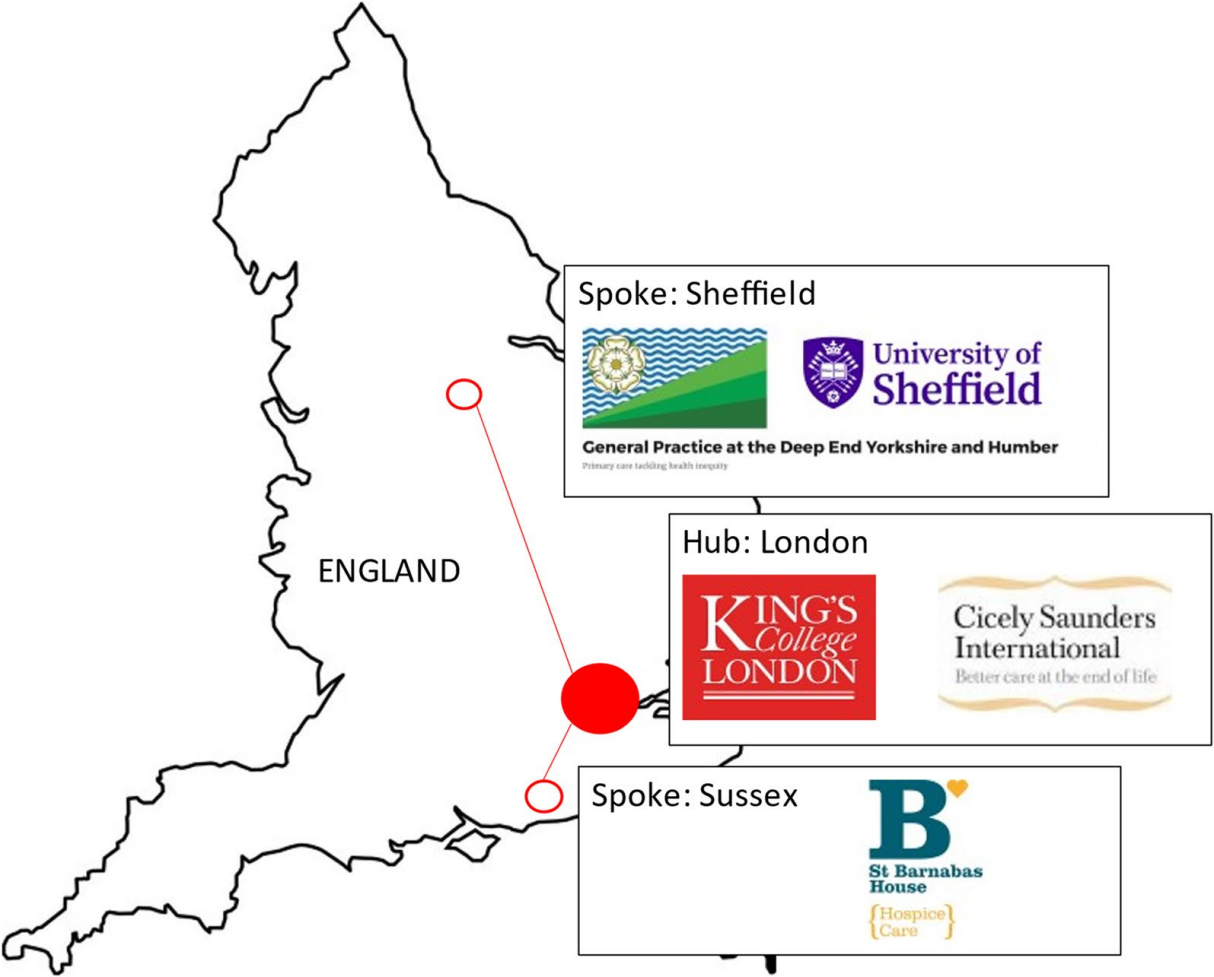
Hub and Spoke model

The RE-EQUIPP Care Partnership brought together a new team of researchers in a “hub and spoke” model (Fig. 1). The Cicely Saunders Institute, King’s College London, acted as the central “hub” because it is a leader in PPI for palliative care, with an established strategy and online forum [13, 14]. The first “spoke” comprised two PPI groups aligned with the University of Sheffield, the DeepEnd Research Alliance, and the Palliative Care Studies Advisory Group. The DeepEnd Research Alliance is a group of researchers working with general practices serving the most socio-economically deprived and diverse areas of the population [15]. The DeepEnd PPI group in Sheffield has been established since 2016 but, until this project, had not been involved in palliative care research. The Palliative Care Studies Advisory Group was formed in 2009, but at the time of this project was coming back together following a break caused by the COVID-19 pandemic. The second “spoke” was St Barnabas Hospice, Worthing, Sussex, serving rural and coastal communities with high levels of deprivation. The PPI group was early in development. The hub and spoke model was intended to enable reciprocal learning and capacity building across both palliative care research and inclusive research based in primary care.

### Design

**Fig. 2:**
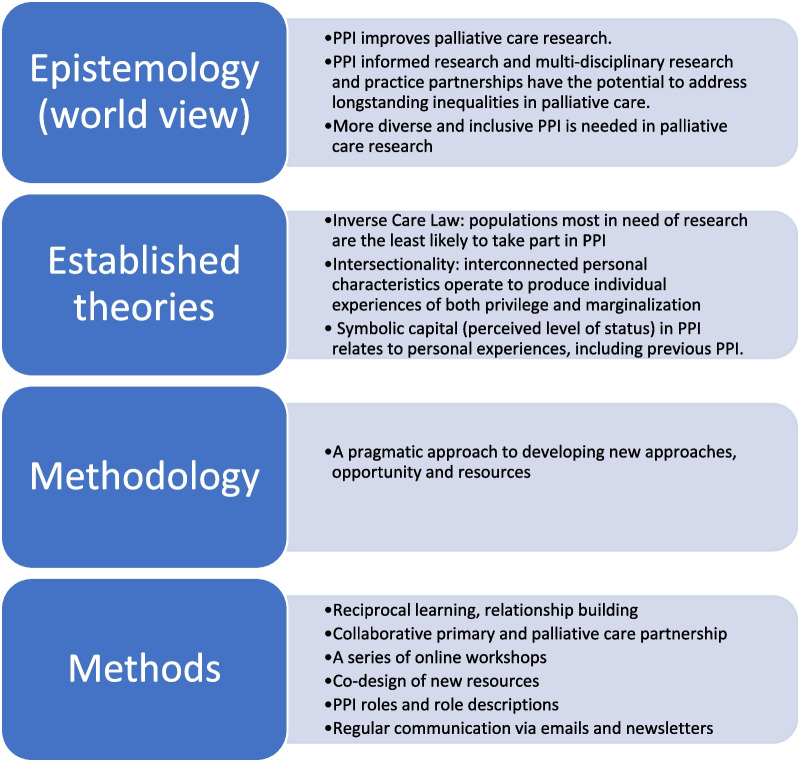
Theoretical framework

The approach taken was developed in close collaboration with PPI members. A theoretical framework (Fig. 2) underpinned a pragmatic, iterative project [16], delivered through a series of workshops, meetings and activities over 12-months. The approach was grounded in the Inverse Care Law, with a commitment to increase the availability of PPI opportunities for people from under-represented and diverse backgrounds [10]. The concept of intersectionality provided a lens through which to consider interconnected characteristics such as race, ethnicity, gender, class, sexuality, culture, religion, social and economic status affecting the individual experiences of both researchers and PPI members of privilege, marginalisation and discrimination [17]. Power dynamics during PPI activities were considered using the concept of symbolic capital (perceived levels of status held by individuals in a social network), with PPI member experiences deliberately prioritised. This challenged traditional power dynamics in PPI, where researchers are the responsible individuals for the project and usual powerholders [18, 19].

Methods were designed to meet the project aims of [1] creating opportunities for more inclusive PPI in palliative care research, and [2] co-designing new resources to support more equitable, diverse, and inclusive PPI for palliative care. Six key principles for effective PPI identified from previous research guided the overall approach and development of a new framework to meet the third aim [20]. An ethical approach was taken, prioritising PPI, maximising benefit for participants and minimising any risk of harm, in accordance with the team’s previous work [21] (Table 1), and the work reported according to the GRIPP2 checklist (included as Additional file: 1) [22].

**Table 1:** An ethical approach to PPI: adapting the approach for this project

Step	Ethical approach	RE-EQUIPP plan
1	Prioritise PPI for palliative care research	All co-applicants and researchers were committed to PPI. Well defined workstream and clear aims for PPI for the partnership were agreed
2	Ensure equity of access to PPI (Justice)	RE-EQUIPP had a specific focus on Equity, Equality, Diversity and Inclusion. Opportunities to extend PPI to people from diverse and deprived backgrounds were devised in collaboration with existing groups, including the University of Sheffield DeepEnd Research Alliance, based in primary care serving the most diverse and deprived populations
3	Agree language and work towards a shared understanding of tasks	Language in palliative care was discussed early and a “jargon buster” created
4	Gain verbal informed consent for PPI	PPI members voluntarily joined the partnership and workshops. Information was provided so that PPI members could make an informed choice about whether to volunteer. PPI members were reminded that they were under no obligation to take part and could stop attending / end their involvement at any point in the partnership work
5	Maximise the benefits for PPI group members	Potential benefits for PPI members included being involved in new learning as the partnership developed, building new networks, opportunity to co—design new resources, take part in presentations, writing papers and future research grant applications
6	Minimise the risk of harm	Each aspect of PPI was designed carefully to avoid potential harm (including emotional distress). Meeting agendas and activities were designed in partnership with PPI members either specifically for an event, and overseen by the Steering Group, where there were two PPI members
7	Provide training for the researcher	Researchers with a range of PPI experiences worked together through the hub and spoke model of the RE-EQUIPP partnership to share experiences and learning
8	Offer training for the PPI group	An informal pre-workshop meeting was held to ensure PPI members felt they were prepared and had the skills to contribute. The workshops were exploratory in nature so no specific training was required
9	Provide funding and recognition	PPI members were reimbursed for their time and expenses with either bank transfers or vouchers, according to their preference

PPI members were identified from existing community networks across the hub and spoke partnership, and nominated by the PPI group facilitators (CE, KF, CG and JB). PPI members were provided with some brief information about the partnership and asked whether they were willing to join the partnership. One member of the research team (NT) co-ordinated the PPI activities. This leadership role was critical to provide a point of contact, to develop plans for activities, reports, and outputs, as well as to liaise regularly with the PPI facilitators from each site. Four workshops and bimonthly steering group meetings took place over 12 months, with regular communication with PPI members via email and newsletters in between these events. PPI activities were designed within a PPI budget for the project, and PPI members chose the level of commitment they were willing to offer. Dates for meetings and workshops were planned early to maximise attendance. Some PPI members required notice to organise carers for an unwell relative. Important festivals were avoided, including Easter and Ramadan.

Prior to the first workshop, two informal online meetings were arranged to share the background and aims of the project. PPI members introduced themselves and started to establish rapport through informal discussion about experiences and knowledge of PPI. A blog, written by one of the experienced PPI representatives (co-author MEO) [23], and the lay summary for the project, were shared with PPI members. These provided a useful starting point for discussions and prompted the idea for a “jargon buster” of frequently used medical terms relevant to the project. This was further developed by the research team and refined by PPI members.

The first workshop was designed to understand barriers and facilitators to PPI, from the perspective of PPI members. PPI members were then involved in the design and conduct of two research workshops (findings reported elsewhere). The workshops focussed on priority-setting in palliative care related to primary care, specialist palliative care and inequalities. Each workshop was led by a member of the research team and supported by an experienced PPI members who opened the workshop with a presentation. Topic guides were used to facilitate open and honest conversations. The fourth and final workshop was designed to test out emerging research ideas with a panel of PPI members. Two research “pitches” (brief research presentations to persuade the panel to support an idea) were presented by research team members, followed by small group discussions. A live scribe (Nifty Fox) captured discussions in real time during the workshop, producing images that summarised the conversations and informed the design of new resources. During the workshops, ideas for resources to support more inclusive PPI in palliative care were captured, and these resources subsequently co-designed with PPI members.

The research team participated in an ongoing process of discussion and peer mentorship throughout the project. Self-awareness and the use of communication skills, including reflecting back and active listening during the workshops, flattened hierarchies and ensured that everyone’s experiences were valued. A “you said, we did” impact log was kept, to record PPI feedback and how that shaped the partnership plan. This informed a PPI progress report that was presented to the steering group at six-monthly intervals.

A short, online questionnaire with free text responses was circulated to PPI members at the end of the project. Questions asked for feedback using a Likert scale to rate the clarity of the project aims and accessibility of information provided. Free text responses were collected regarding personal experiences, ability to contribute during the workshops and whether PPI members felt their contribution was valued.

### Outcomes

In total, 16 PPI members joined the project, eight from the Cicely Saunders Institute group, two from Sussex and six from Sheffield (four from the DeepEnd group and two from the Palliative Care Studies Advisory Group). Most were White British (*n* = 10), other ethnicities were Asian (*n* = 4), Black African (*n* = 1) and British mixed race (*n* = 1). There was a range of experience in terms of PPI for palliative care. Members from Cicely Saunders and the Sheffield Palliative Care Studies Advisory Group all had prior experience. Members from the DeepEnd group and Sussex had not participated in PPI for palliative care research before, however all had relevant personal experience of receiving palliative care personally or caring for a relative.

All attended the first workshop about PPI for palliative care in May 2022, following which four PPI members agreed to attend the research prioritisation workshops in July and September 2022, and two volunteered for project steering group membership. The final workshop was held with all 16 PPI members in November 2022. A total of eight PPI members provided feedback via the online survey, of whom 80% (6/8) agreed or strongly agreed that the aim of the workshops was clear, and that the information provided was adequate. Qualitative feedback was limited, but grouped into two broad overarching areas (positive feedback and learning) with illustrative quotes provided in Table 2:

**Table 2:** Evaluation feedback and learning

	Area of feedback	Illustrative quotes
Positive	Clarity of aims	*“The quality of the information, the presentations and the way it was designed to involve everyone”*
	Feeling valued and able to contribute	*“The enthusiasm, the fact that we are all heard and listened to. Mostly, the fact that something we suggest is being taken up”*
	Accessibility and diversity of the group	*“The rainbow of insights and experience of the group. It will help the medical community put together the best possible plans for the project, we were discussing. It came out, what the needs of patients are. The small suggestions that came out, that can make such a huge difference”*
Future learning	Further preparation for the workshops would clarify aims and enable more involvement	*“Maybe more reading material beforehand to really absorb the breadth of initiatives taking place”*
	Workshops could be improved with clearer structure and objectives, so that PPI members could understand the expectations of the research team and contribute more effectively	*“Workshops were well managed and respected, but these could have been more structured and prepared with key objectives and outcomes clearly communicated both in advance of the workshop and immediately after the event (within 2 or 3 weeks maximum)”*
	Follow-up after the workshops is necessary	*“There was so much to discuss, and we often ran out of time during the breakout sessions. Would love to be updated about progress”*

There was an active process of self-reflection and learning within the research team throughout the partnership. The team were open to contributions that challenged their existing views, expectations, unconscious biases, and traditional power dynamics. The use of commonly used medical phrases related to palliative care, primary care, and research was also challenged. The challenge of ensuring that language is accessible was discussed in detail, and the jargon buster created to address this.

PPI members held status (symbolic capital) in interactions, where their opinions, personal experiences and diversity were deliberately prioritised. The importance and relevance of equity and justice were advocated by a PPI member (and co-author) J Beng in both PPI and future research. Open discussions took place about individual lived experiences of serious illness, healthcare services, socio-economic deprivation, and racism. The nature of the conversations meant that PPI members felt able to provide challenge, and racism was specifically discussed as a barrier to inclusive PPI and research. Researchers were challenged to consider their own status, personal characteristics, and experiences of privilege and / or discrimination in the interactions throughout this work. Specifically, researchers were challenged around “White British models of palliative care that did not work for all communities”. PPI members described lived experiences of racism and problematic cultural norms in healthcare. There was openness about the status and position of some group members related to prior experience of research and PPI activities (or not), and valuable insights into how much some PPI members value the sense of purpose provided by their involvement. The importance of timely reimbursement was a particularly pertinent issue for PPI members who had given up regular employment to become carers for people with palliative care needs.

**Fig. 3:**
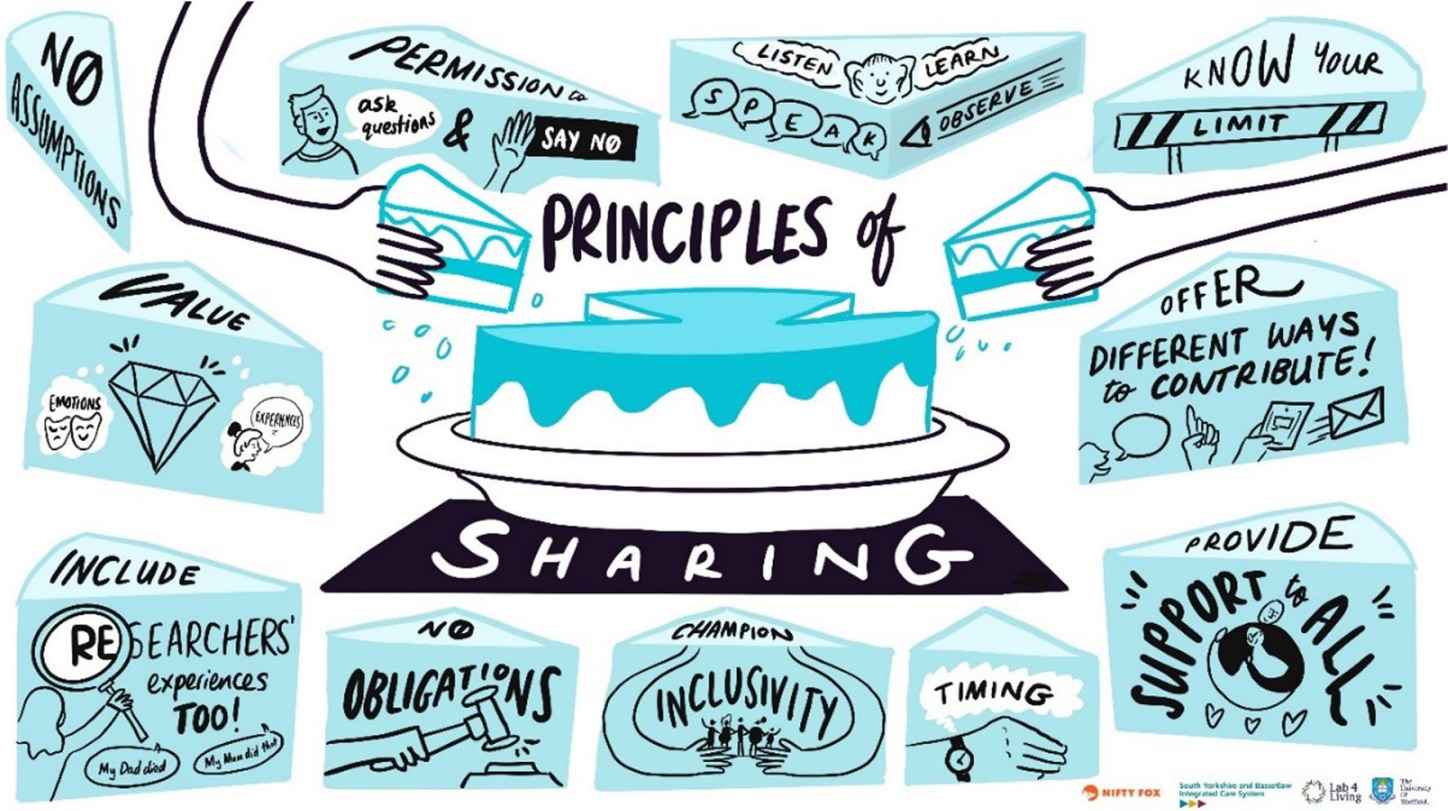
Principles of sharing in PPI for Palliative Care

**Fig. 4:**
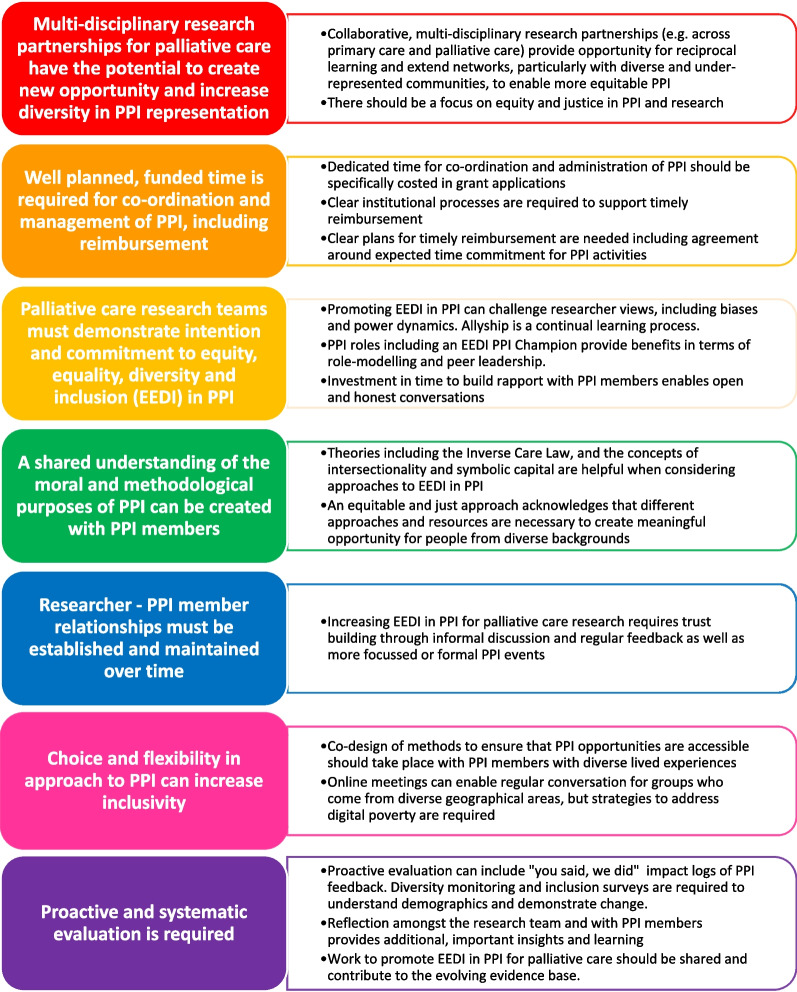
A framework for Equity, Equality, Diversity and Inclusion (EEDI) in PPI for palliative care research

Resources developed through this project to support more inclusive PPI for palliative care in the future are:

1. The “jargon-buster” (Additional file: 2) to provide clarity around frequently used medical terms relevant to this project.
2. A PPI flyer outlining the role and purpose of PPI, and a role description for RE-EQUIPP (Additional file: 3) to help with recruitment of new PPI members.
3. A role description for a PPI Equity, Equality, Diversity, and Inclusion (EEDI) Champion (Additional file: 4), specifically intended to provide one of the PPI members (co-author) J Beng with a position from which he could challenge the research team, hold them to account for inclusive approaches, and provide peer leadership and role-modelling.
4. Accessible resources and information for PPI, including the use of images in a comic format, and a short, animated video, including:
  An online, open access “recipe book” guide for both PPI members and researchers, outlining the definition and purpose of PPI, likely format of meetings and what to expect. This is hosted on a project website.
  A new PPI in palliative care animation, aimed at people who might be interested in becoming PPI members, hosted on the project website [add website]. A “principles of sharing” graphic, highlighting factors for both researchers and PPI members to consider in PPI work such as the importance of listening, being able to stop contributing if the session was uncomfortable with no obligations, and the need to agree a variety of ways to contribute (in person, online, by email) to improve inclusivity (Fig. 3):

A new framework for equitable PPI in palliative care research was devised (Fig. 4), drawing upon the learning from the project, principles for PPI that informed the work [20], theoretical underpinnings, multi-disciplinary partnership working and innovations including the new co-designed resources. The framework is intended to inform PPI for future palliative care research, outlining the need for research team commitment and shared vision, adequate resource, careful planning, relationship building and evaluation.

## Discussion

This paper reports the PPI conducted through the RE-EQUIPP Care Partnership. The aims of the project, to create opportunities, co-design new resources and propose a new framework for more equitable and inclusive PPI in palliative care research were met through a new collaborative partnership across primary care and palliative care in three areas of the UK. The project outputs and resources are intended to inform more equitable, diverse, and inclusive PPI for palliative care research in the future. Shared commitment to increasing diversity in PPI was necessary across the “hub and spokes” of the partnership. PPI members were recruited from both primary care and palliative care research networks, bringing together a group with diverse personal characteristics and experiences.

### Strengths and limitations

A strength of this work was the agile, responsive, pragmatic approach to PPI, with clear aims met through reciprocal learning from a multi-disciplinary collaboration of researchers, in partnership with PPI members. The partnership project brought together a team of researchers and PPI members who were highly committed to new approaches to improve inclusivity in PPI. This required researchers to be willing to accept challenge and reflect on their own position in interactions. The approach was informed by good practice principles from previous PPI, the Inverse Care Law and a theoretical framework that referred to symbolic capital and intersectionality. The research team were committed to ensuring that people who were least likely to take part in PPI for palliative care research were proactively provided with the opportunity to do so. There was shared understanding that opportunities must be proactively created for people from diverse and under-represented backgrounds to take part in PPI. Trusted relationships were established, allowing open conversations and a continual process of evaluation and learning, including self-reflection amongst the team.

This work has several limitations. Despite efforts to recruit PPI members from diverse backgrounds, the majority were White British. All meetings were conducted online, which had the benefit of bringing together a group from diverse geographical areas of the UK, but future work must also consider the most effective ways to involve PPI members who experience digital poverty.

In terms of recording and co-ordinating the PPI work, opportunities to collect more detailed demographic information from PPI members were missed. This is important to consider in future PPI and research. Diversity monitoring surveys capture more demographic information would be helpful at the start and end of projects. Inclusion and engagement surveys provide more opportunity to understand barriers to participation and perceptions of culture. This paper offers some ideas around improving diversity and inclusion, but much more work is needed. In this potentially sensitive area, researcher’s must be open and honest, avoid tokenism, and be prepared to learn from mistakes.

Clarity around reimbursement processes were necessary, to ensure timely reimbursement, which is pertinent for PPI members from socioeconomically disadvantaged areas. Agreeing the expected time commitment for activities with PPI members is prudent to avoid conflict or overspend of limited PPI budgets. Researcher time to develop learning outputs, draft papers for publication and for the development of new PPI resources was limited by the funding available for the project. As the PPI work grew, it was necessary to balance the ambition of the team with the potential to over-burden PPI members and available time and financial resource.

### Comparison with existing literature

People from minoritised communities, who often have the highest health need, are under-represented in research [24]. Inclusion and exclusion criteria for research may actively exclude people from more diverse backgrounds. This is a pertinent issue in all research. In palliative care, recommendations from research have the potential to inadvertently cause harm to those who are not represented [25, 26]. Existing tools and frameworks for planning and reporting PPI for palliative care (the Public Involvement in Research Impact Tookit: PIRIT [27]) and PPI more widely (Guidance for Reporting Involvement of Patients and the Public: GRIPP-2 [22]) reference the need for inclusive opportunities with PPI members that reflect the population of research interest and a clear description of the people involved with PPI activities, but currently there is a lack of guidance for how this can be achieved.

While there were limitations to this project, it does represent progress in improving equity, equality, diversity, and inclusion in PPI for palliative care research. The learning and resources from the project are intended to inform future PPI in this field and can be further developed in the future. Specific considerations include recruitment of PPI members to research to ensure diverse and equitable representation. Successful strategies to recruit PPI members include working with community organisations and community leaders to increase discussion and awareness of research, and address longstanding suspicion and mistrust [28].

This project was cognisant of traditional power dynamics in PPI activities and interactions. The partnership placed equal emphasis on the contributions of researchers, clinicians, and PPI members, however some final decisions about the partnership were made by researchers. PPI that avoids tokenism should include strategies for PPI members to co-create ideas, hold power in carrying out the research, have delegated responsibility for project resources and direct involvement in decision-making [28]. Researcher-clinician-PPI member partnerships require openness, honesty, regular evaluation, and reflection to ensure these goals are met.

In PPI for palliative care research, where the subject area is potentially distressing, an important future consideration is support for PPI members, through debrief or extra informal meetings. These need to be easily accessible and could be provided by someone who is not directly involved with the project, to overcome researcher-PPI member power dynamics. An “open door” session and / or an informal post-workshop meeting may be helpful. The potential value, purpose, and management of peer support for PPI members through “buddying”, for example via an email group, online forum, or messaging service also warrants further attention [14]. Ethical issues related to buddying in PPI for palliative care research, including the risk of distress from bereavement, require careful consideration.

Much more research is needed to understand and address the intersection between ethnicity and other social determinants of health and inequalities in palliative care. Recent reviews call for proactive change, with decolonisation of research teams to overcome processes that are commensurate with racism in palliative care research [25]. Improving equity, equality, diversity, and inclusion in both PPI and palliative care research in the future will require clear focus on the concepts of equity and justice and recognition that people from diverse communities want the opportunity to be involved. Clear evaluation and reporting will ensure shared learning and development of best practice. Allyship, defined as “support for and practice of promoting rights, representation and inclusion by members of an advantaged group to advice the under-represented or marginalised” is an important and continual learning process [29, 30] and should be adopted by researchers and clinicians as they undertake future work.

## Conclusion

More inclusive approaches to PPI in palliative care are necessary to inform research and improve palliative care in the future. Multi-disciplinary partnerships can create opportunities through reciprocal learning, innovative approaches, and co-designed resources to increase inclusivity and diversity in PPI for palliative care research. The evidence base in palliative care PPI is under-developed and this risks the integrity of PPI activities. This work contributes to this evolving evidence base by presenting a new framework for PPI in palliative care research, intended to provide practical guidance for researchers wishing to improve inclusivity and diversity in their work. The framework highlights the need for research team commitment and shared vision, adequate resource, careful planning, relationship building and evaluation.

### Supplementary Information

**Additional file 1.** A framework for improving patient and public involvement opportunities for research to address inequalities in palliative care. GRIPP2 checklist (long form).

**Additional file 2. **Jargon Buster for primary care and palliative care.

**Additional file 3. **Patient and public involvement member role description.

**Additional file 4. **Equity, equality, diversity and inclusion champion role description.

## Data Availability

[add website when ready]. The manuscript does not contain research data.

## Abbreviations

PPI: Patient and Public Involvement
RE-EQUIPP Care: REducing inEQUalities through Integration of Primary and Palliative Care
