# Supplementary material for: A framework for more equitable, diverse, and inclusive Patient and Public Involvement for palliative care research
Source: Res Involv Engagem. 2024 Feb 8;10:19. doi: 10.1186/s40900-023-00525-3 (PMC10851547; doi:10.1186/s40900-023-00525-3)
Supplement: Supplementary file 2 — Additional file 2. Jargon Buster for primary care and palliative care. [file 40900_2023_525_MOESM2_ESM.docx]

# Supplementary file 2: Jargon Buster for primary care and palliative care

| **Speciality** | **Definition** |
| --- | --- |
| **Primary care** | The first place that people go to for health advice and treatment in the community. It includes disease prevention and treatment, living with long term conditions and palliative care.  Primary care includes a wide range of professionals including community nursing services, therapists, opticians, pharmacists, and dentists. General practitioners are medical specialists in primary care. |
| **Palliative care** | A broad approach to care that is person-centred and focussed on quality of life for people with serious illness, and their carers. |
| **End-of-life care** | Care for people with advanced disease who may be in the last year of life. |
| **Specialist palliative care** | Palliative care delivered by professionals with qualifications, expertise, and experience in palliative care, for people with complex needs that cannot be met by their current care team. |
| **Multidisciplinary team (MDT)** | A group of health and care practitioners from different organisations (e.g. GPs, social workers, nurses), that work together to make decisions regarding the treatment of individual patients. |
| **Person-centred care** | Care focussed on the needs of an individual, to ensure the person’s preferences, needs and values guide clinical decisions. |
| **Holistic care** | Care and treatment of the whole person, that considers physical, mental, social, cultural factors and spiritual beliefs, rather than just the symptoms of an illness. |
| **Integration** | The process of combining two or more teams or organisations into one. |
| **Collaboration** | Two or more people, teams or organisations working together to complete a task or achieve a goal. |
| **Health inequalities** | Avoidable and unfair differences in health between different groups of people. |
