# Supplementary material for: A framework for more equitable, diverse, and inclusive Patient and Public Involvement for palliative care research
Source: Res Involv Engagem. 2024 Feb 8;10:19. doi: 10.1186/s40900-023-00525-3 (PMC10851547; doi:10.1186/s40900-023-00525-3)
Supplement: Supplementary file 3 — Additional file 3. Patient and public involvement member role description. [file 40900_2023_525_MOESM3_ESM.docx]

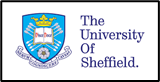

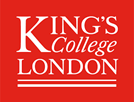


**
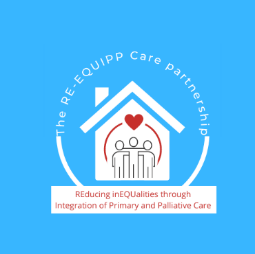
The RE-EQUIPP Care Partnership: REducing inEQUalities through Integration of Primary and Palliative Care**

**Patient, Family Member or Carer Role**

Thank you very much for your interest in joining the RE-EQUIPP care partnership as a patient, family member or carer. This document provides you with further information about the partnership, and how you can help.

# Who are we?

We are a group of researchers and patients, family members or carer representatives with experience of palliative care.

We have been given some funding by the National Institute for Health Research. We want to use this to work out what the most important issues are for us to research, bringing together primary and palliative care.

We want to put patient and public views at the centre of this work.

Primary care is the first place in the community most people go to for healthcare advice and treatment. It includes general practices (GPs), district nurses and pharmacists.

Palliative care helps people who have a serious illness by providing relief from the symptoms and stress of the illness. The goal is to improve quality of life for both the patient and the family.

Primary care has an important role in giving palliative care to people in the community, alongside specialist palliative care services.

Research is important in helping us to understand what works best. However, there is not much research which looks at palliative care and primary care together.

This research partnership brings together world-leading experts in palliative care research from the Cicely Saunders Institute, King’s College London, with primary care research in two other areas of England: (1) diverse, inner-city Sheffield in South Yorkshire, and (2) the rural area of Sussex.

# What is the purpose of this role, and who can be involved?

When developing and doing research, it is important to us that we understand patients’, families’ and carers’ needs, views, and priorities.

This helps to ensure that the research is in the best interests of patients and their families. Also, it helps our research be as useful, accessible, and meaningful as possible.

For that reason, we want to invite people with personal experience of palliative care as patients, family members or carers to work with us on our RE-EQUIPP care partnership.

# What is the project?

The RE-EQUIPP care partnership aims to:

1. Build a partnership between primary and palliative care to do research which leads to better palliative care in the community,
2. Lead and develop new research into how primary and palliative care services can work together (integrate) better to ensure everyone gets good palliative care,
3. Put patients and the public at the heart of the partnership, and
4. Apply for further funding to develop new ways of delivering palliative care for people in the community.

# How can you help?

We appreciate that due to other commitments people may not be able to do everything.

Below is a list of tasks you may wish to take part in.

Please tick any tasks that you would like to be involved in.

Some of these things may be new to you – but don’t worry, you don’t need to know anything about research, and support is available.

We are interested in your experience of palliative care, and what you think.

Attend meetings to help shape the direction of the RE-EQUIPP care partnership

Attend workshops and events involving the researchers, invited academics and the public

Share your experiences of receiving palliative care, and/or of caring for someone with advanced illness.

Ensure that the project responds to views of local patients, families and/or carers.

Offer advice on how best to involve patients, families and/or carers in our research.

Support other patients, families and/or carers to be involved in the RE-EQUIPP care partnership.

Help to decide what is important for future research.

Provide feedback on proposed research and/or comment on study materials (e.g. patient information sheets).

# What do we require from you?

# Please tick all that apply:

Willingness and availability to attend meetings/events online using Google Meet and/or Zoom (reasonable costs will be reimbursed). We can help you to get online if necessary.

Willingness to actively contribute to meetings, listen to others, and tell your story as you feel able.

Work well with others in teams, networks and organisations.

Try to take an objective view, seeing issues from different perspectives.

Question information and explanations supplied by others (e.g. researchers) when they are not clear.

Respect confidentiality requirements and declare any conflicts of interest.

# When and where will we meet?

Meetings will be held online using Zoom.

You will be sent a link to the meeting along with an agenda and any documents by email before each meeting.

The RE-EQUIPP care partnership will be meeting once every two months until the end of January 2023.

There are three workshops planned for dates in May, June and July 2022.

You do not have to take part in every meeting or workshop or remain involved until the end of the partnership if you do not wish to.

# Confidentiality and Conflict of Interest

You will be asked not to share confidential information you may receive as a result of your membership of the RE-EQUIPP care partnership.

You will be required to tell us about any involvement you may have with other organisations, government bodies or corporate/commercial interests, which could result in a conflict of interest with the work conducted by the RE-EQUIPP care partnership.

# How can we support you in the role?

We will be available to provide you with guidance and answer any questions throughout the project – of course your ideas and suggestions are also welcome.

We will support you to contribute to the partnership.

You will receive a payment of £25 per hour for your time, expertise and involvement for attending any RE-EQUIPP care partnership meetings in line with the National Institute for Health Research guidelines:

<https://www.nihr.ac.uk/documents/payment-guidance-for-researchers-and-professionals/27392>

Payment will be in cash by bank transfer, or in vouchers – you can choose what is best for you.

What if I have questions?

If you have any questions about the project or your role or for any more information about the advertised opportunity, please contact ***[insert name***] by telephone ***[insert telephone number]*** or email: ***[insert email address]***.
