# Supplementary material for: A framework for more equitable, diverse, and inclusive Patient and Public Involvement for palliative care research
Source: Res Involv Engagem. 2024 Feb 8;10:19. doi: 10.1186/s40900-023-00525-3 (PMC10851547; doi:10.1186/s40900-023-00525-3)
Supplement: Supplementary file 4 — Additional file 4. Equity, equality, diversity and inclusion champion role description. [file 40900_2023_525_MOESM4_ESM.docx]

**RE-EQUIPP Patient and Public Involvement**
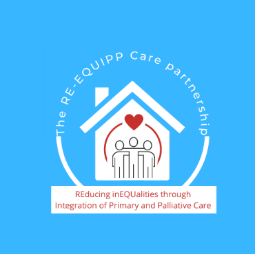


**EQUITY, EQUALITY, DIVERSITY AND INCLUSION CHAMPION ROLE DESCRIPTION**

What is EEDI?

- **Equity:** is where all people are provided the same opportunity, irrespective of their status and identity.
- **Equality:**is about equal access to opportunities, removing barriers and disadvantage for everyone.
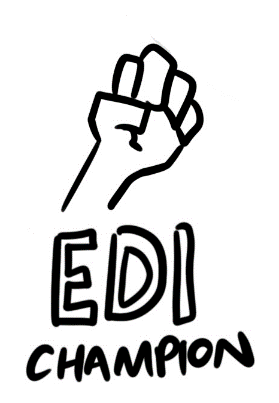

- **Diversity:**is about being reflective of the wider community and supporting representation.
- **Inclusion:**is about valuing, respecting, and celebrating difference and building a sense of belonging.

What will the EDI Champion do?

- ensure that EDI matters are raised and discussed at project meetings and workshops
- be prepared to constructively challenge the EDI dimension of partnership decisions and plans
- review partnership documents and publications from an EDI perspective
- be a point of contact for others who wish to raise EDI issues or concerns
- encourage and support others to speak up on issues or concerns they may have
- keep a record of their experience in the role so that it can be evaluated

What does the EDI Champion need?

- an awareness of issues relating to equality, diversity and inclusion
- a commitment to promoting EDI within the RE-EQUIPP partnership
- listening skills
- effective communication skills and an approachable manner
- ability to be objective
- team working skills
- willingness to contribute to problem-solving

What support do we offer?

- a named contact on the RE-EQUIPP team who can provide advice and support
- reimbursement for time taken on the EDI Champion role
- support to network with other partnership members interested in promoting EDI
